# Imaging of carbon nanomembranes with helium ion microscopy

**DOI:** 10.3762/bjnano.6.175

**Published:** 2015-08-12

**Authors:** André Beyer, Henning Vieker, Robin Klett, Hanno Meyer zu Theenhausen, Polina Angelova, Armin Gölzhäuser

**Affiliations:** 1 Physics of Supramolecular Systems and Surfaces, Bielefeld University, 33615 Bielefeld, Germany; 2 CNM Technologies GmbH, 33609 Bielefeld, Germany

**Keywords:** 2D materials, carbon nanomembrane, helium ion microscopy, self-assembled monolayers

## Abstract

Carbon nanomembranes (CNMs) prepared from aromatic self-assembled monolayers constitute a recently developed class of 2D materials. They are made by a combination of self-assembly, radiation-induced cross-linking and the detachment of the cross-linked SAM from its substrate. CNMs can be deposited on arbitrary substrates, including holey and perforated ones, as well as on metallic (transmission electron microscopy) grids. Therewith, freestanding membranes with a thickness of 1 nm and macroscopic lateral dimensions can be prepared. Although free-standing CNMs cannot be imaged by light microscopy, charged particle techniques can visualize them. However, CNMs are electrically insulating, which makes them sensitive to charging. We demonstrate that the helium ion microscope (HIM) is a good candidate for imaging freestanding CNMs due to its efficient charge compensation tool. Scanning with a beam of helium ions while recording the emitted secondary electrons generates the HIM images. The advantages of HIM are high resolution, high surface sensitivity and large depth of field. The effects of sample charging, imaging of multilayer CNMs as well as imaging artefacts are discussed.

## Introduction

Carbon nanomembranes (CNMs) are extremely thin and homogeneous two-dimensional objects consisting of a monolayer of laterally cross-linked molecules. They are made by exposing a self-assembled monolayer (SAM) of aromatic molecules with electron [1] or soft X-ray irradiation [2], which results in the cross-linking of neighbouring molecules into a CNM of molecular thickness. The CNM is then released from its substrate by dissolving the latter [3]. The thickness, chemical composition, and density of the original SAM determine the mechanical properties, such as elasticity and porosity, as well as the chemical composition of the resulting CNM. The freely suspended CNMs are made by transferring the cross-linked SAM from its substrate to a holey structure, such as a metal grid. The resulting CNM is approximately as thick as the original SAM and can span macroscopic areas; thus far, freestanding CNMs of up to 0.5 × 0.5 mm^2^ have been fabricated.

The electrical conductivity of the CNM can also be tailored, as pyrolysis results in a gradual transformation into graphene [4–6]. CNMs have potential for many technical applications, such as filters [7], sensors [4], resists [8], nanosieves [9], or “lab-on-a-chip” devices [10]. Many aspects regarding the fabrication, modification and functionalization of homogenous as well as patterned CNMs are compiled in a recent review [11].

Optical microscopy is suitable for imaging CNMs on SiO_2_/Si wafers [12], but on other substrates, CNMs are not (or only barely) visible. In particular, it is not possible to directly image freestanding CNMs by regular optical microscopy. Indirect optical methods require the attachment of particles, fluorescent dyes [13], metallic nanostructures [14] or other suitable indicators that are detectable by optical microscopy. In addition, optical imaging with a Mirau interferometer allows the detection of the vibrational modes of bare CNMs with a resolution limited by the light wavelength [15].

The imaging of CNMs with higher magnification requires charged particle microscopy techniques such as scanning electron microscopy (SEM) or helium ion microscopy (HIM). As illustrated in Supporting Information File 1, Figure S1, SEM shows a low signal-to-noise-ratio for freestanding CNMs, especially at higher magnifications, due to charging issues [4,16]. This tends to be destructive for freestanding membranes. For example, an attempt at imaging perforated CNMs with SEM failed due to charging-induced rupture during the imaging process [9]. On the other hand, HIM is very well-suited to image CNMs with high signal-to-noise-ratio at high magnification. In this report, we will show examples that support this statement. We demonstrate the effect of charging on HIM images as well as the effectiveness of the HIM charge compensation mechanism. The principle of operation of HIM as well as a recent overview of HIM-related reports can be found elsewhere [17]. In short, HIM utilizes a focussed beam of He^+^ ions that scans the sample surface. The image is usually obtained by the detection of secondary electrons. The imaging of insulating samples may lead to positive charging due to the emission of secondary electrons as well as the exposure to positive He^+^ ions. A major advantage of HIM is its ability to compensate for sample charging by employing an electron flood gun in an alternating manner. In this way, the sample is exposed to electrons between scans of subsequent image lines or frames.

There is scarce literature on HIM imaging of ultrathin membranes. Many researchers have examined graphene, where the main focus was on the modification and production of small structures and circuits [18–22]. The thickness of graphene is comparable to CNMs, but a fundamental difference is its high conductivity, which eases charged particle imaging. Small flakes of hexagonal boron nitride (h-BN), an insulating material that shares similarities with graphene, were imaged in a comparative study [23]. Therein, it is shown that HIM is more sensitive and consistent than SEM for characterizing the number of layers and the morphology of 2D materials. It was also shown that HIM is very sensitive in characterizing supported, thin organic layers due to its high surface sensitivity [24–25].

## Results and Discussion

**Figure 1:**
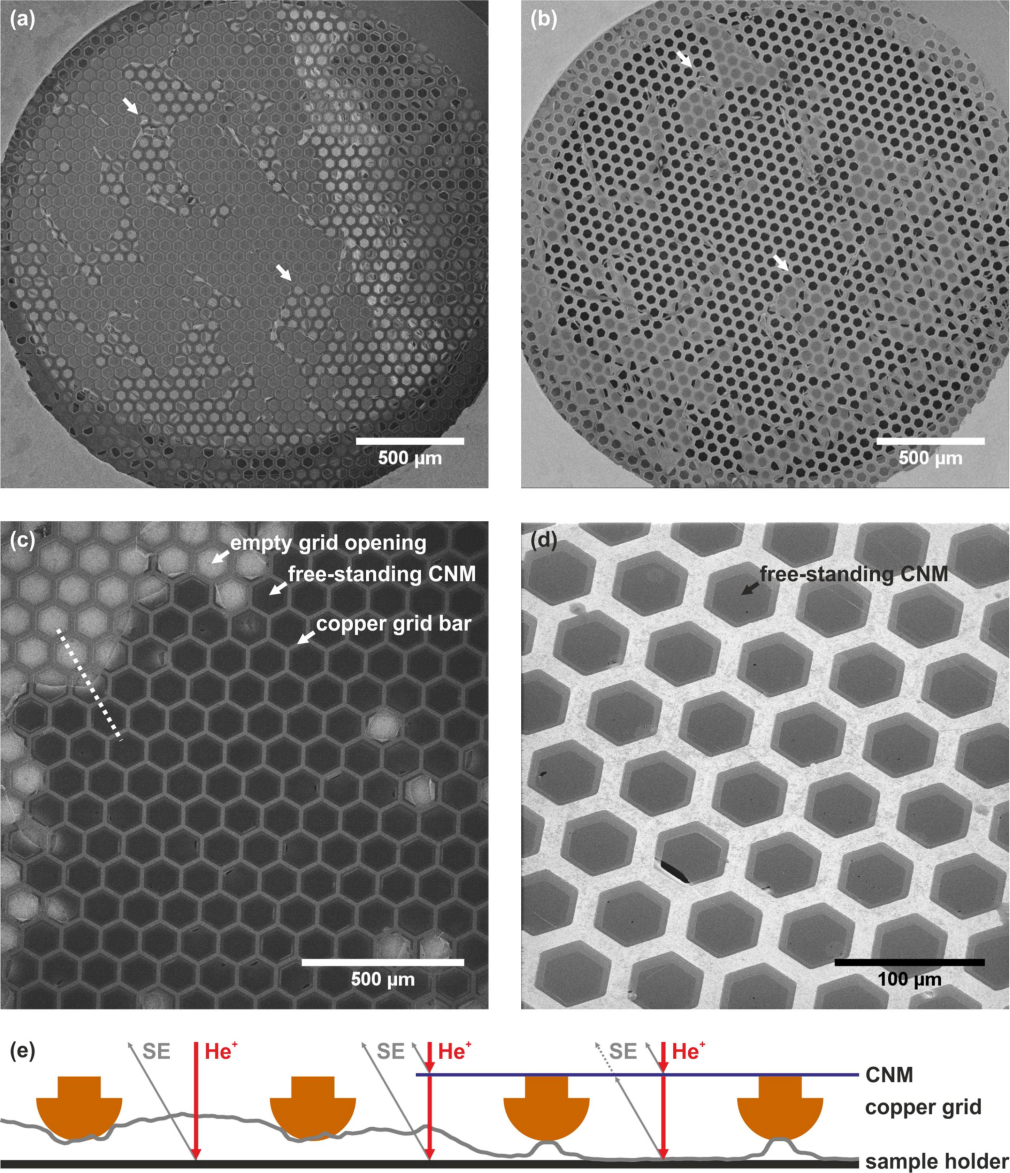
(a, b) HIM images of freestanding CNMs on TEM grids, illustrating the importance of the background. Both images show the same sample mounted differently. The arrows point to the same positions as a guide to the eye. (c) CNMs on a TEM grid with a bright background and substantial membrane charging. (d) CNMs are imaged on a dark background with negligible membrane charging. (e) Schematic cross-section and superimposed line profile of the image greyscale values along the dotted line in (c) with the primary He^+^ beam and secondary electrons emitted from the CNM and the sample holder depicted at three exemplary locations. The values of the line profile (grey curve) are a measure of the amount of detected secondary electrons. Detailed information on all HIM images are given in Supporting Information File 1, Tables S1 and S2.

For imaging with the HIM, the most important characteristics of CNMs are that they are ultrathin (≈1 nm) and electrically insulating. Due to the low thickness, the high surface sensitivity of the HIM is well suited to obtain CNM images with high signal-to-noise-ratio. It is also important to note that the helium beam easily penetrates the CNM and also strikes objects below the freestanding membrane, for example, the sample holder. Figure 1 shows an example of this effect. The images in Figure 1a,b show the same sample: a hexagonal TEM grid is mounted in a sample holder (visible in the four corners of the images) which has a mm-sized, circular opening. The CNM partly covers the TEM grid and the white arrows indicate CNM-covered regions. Although both HIM images were taken with the same ion acceleration voltage and similar ion currents, the contrast in the images appears almost inverted. This difference relates to the background: In Figure 1a, the main part of the grid is placed closely over the homogeneous metal surface of the sample holder. An edge of the sample holder surface is visible as a bright strip running from the top to the lower right of the image. These background features are visible in the HIM image as He^+^ ions impinge upon the sample holder behind the grid and eject secondary electrons that reach the SE detector without being blocked. In Figure 1b, the sample holder background is not visible as the path of the secondary electrons emitted from the sample stage to the detector has been blocked by mounting the grid on top of a deep cavity, which acts like a Faraday cup. Thus, in Figure 1b, the uncovered openings of the grid appear dark in all parts of this image. To guide the eye, white arrows in Figure 1a,b depict the same position in the sample. Note that regardless of the CNM grid mounting, in both cases, a fast evaluation regarding the area of intact CNMs is easily obtained due to the large field of view (of more than 2 mm), high depth of view, and high contrast between bare and CNM-covered grid meshes. The recording time of such images is less than one minute.

Another effect, which substantially changes the appearance of the CNM image, is electrostatic charging of the ultrathin, insulating membranes. In Figure 1c,d HIM images with and without charging artefacts are compared. A schematic cross-section of the sample as well as a superimposed line profile of the image greyscale values in Figure 1c corresponding to the white dotted line is given in Figure 1e. An empty grid opening on the left is followed by a partial and a fully covered opening. CNM charging due to the positively charged He^+^ ion beam and the emission of negatively charged secondary electrons can only result in positive charging regardless of the secondary electron yield of the CNMs. A positively charged sample will hinder the emission of secondary electrons. Therefore, positively charged CNMs will appear dark in HIM images. This is observed in Figure 1c where the freestanding regions of the membranes are dark, while the membrane regions directly in contact with the copper grid appear much brighter. In the latter, secondary electrons are also emitted from the underlying copper grid and charges in the CNM are neutralised by the metallic support. This combination of effects yields a high contrast between the CNM-covered and non-covered regions. However, the structural details of the CNMs cannot be investigated under such imaging conditions. An interesting image feature appears in partially covered meshes: the edges of freestanding CNMs are brighter than intact CNMs, as illustrated in the area near the centre of the dotted line. This effect is explained by considering that secondary electrons are emitted from the sample support rather than from the freestanding CNMs itself, as schematically depicted in Figure 1e. The intact CNMs completely block the path of such secondary electrons to the detector while partially ruptured CNMs do not.

The reduction of the beam current, the dwell time per pixel, the use of frame averaging as well as charge compensation can reduce or completely avoid the charging of insulating membranes. These imaging parameter changes resulted in Figure 1d, which does not show any notable charging effects. Here, the sample was mounted in a way that no secondary electrons from the sample holder could reach the detector. A small rupture in the CNM reveals a high contrast between the bright CNM and the dark background. Under these imaging conditions, fine details on the top of freestanding CNMs can be observed. For example, small pores and folds are visible.

**Figure 2:**
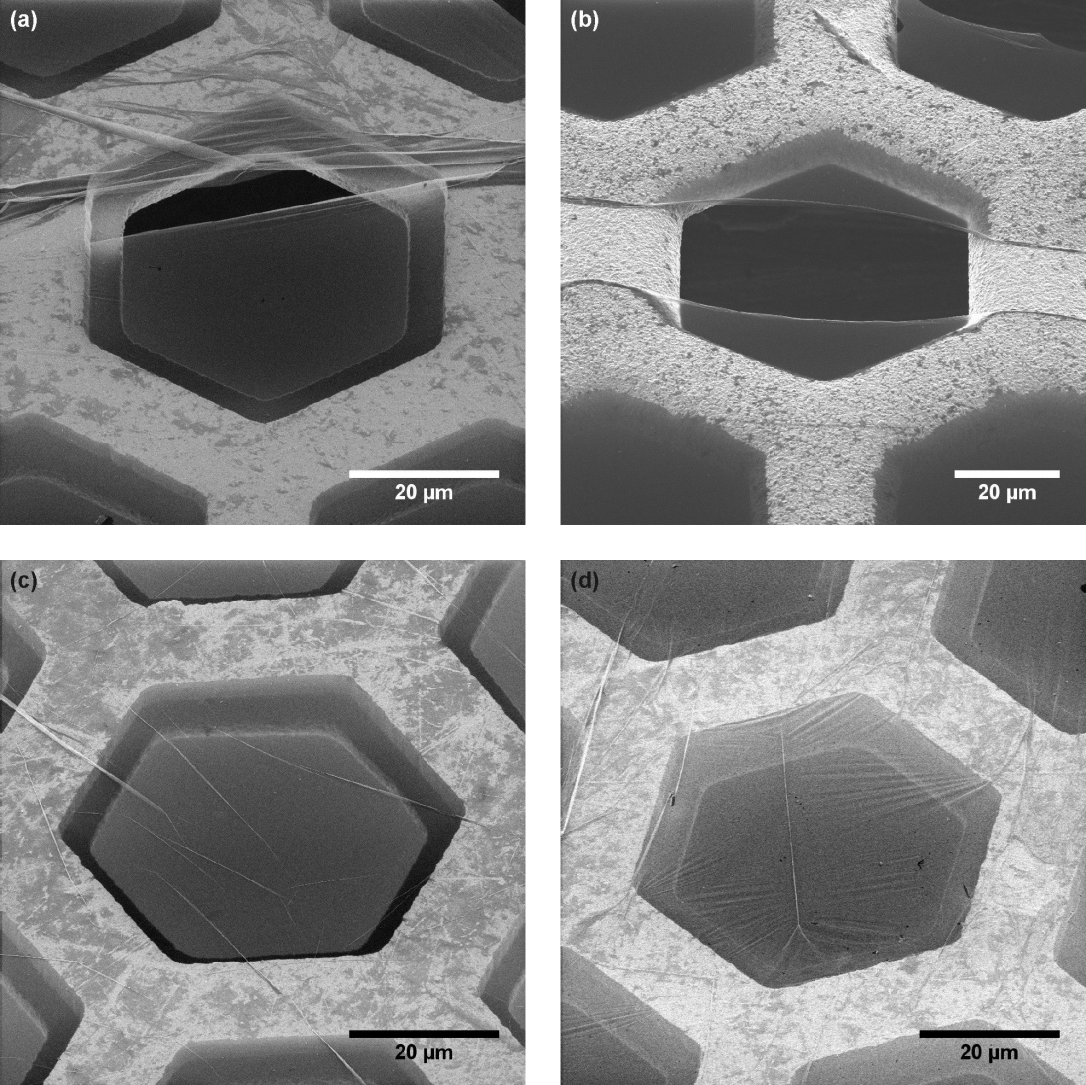
Examples of CNMs which were transferred onto copper grids with hexagonal openings. Different types of features are visible: (a) larger folds; (b) rolled CNM edges; (c) small folds; and (d) wrinkled CNMs. Detailed information on all HIM images is given in Supporting Information File 1, Tables S1 and S2.

A collection of different CNMs on hexagonal copper grids is presented in Figure 2, exhibiting the different types of features that are visible in HIM images. From these images, one intuitively obtains an impression of the detailed shape of the copper grid and the CNM on top. In Figure 2a larger folds on the upper side of the image and one rupture in the centre are visible. Figure 2b is an example of a membrane rolling up at a rupture, showing the high flexibility of CNMs. Small folds like those in Figure 2c are frequently observed, while wrinkling of the freestanding membrane (Figure 2d) is less often observed.

**Figure 3:**
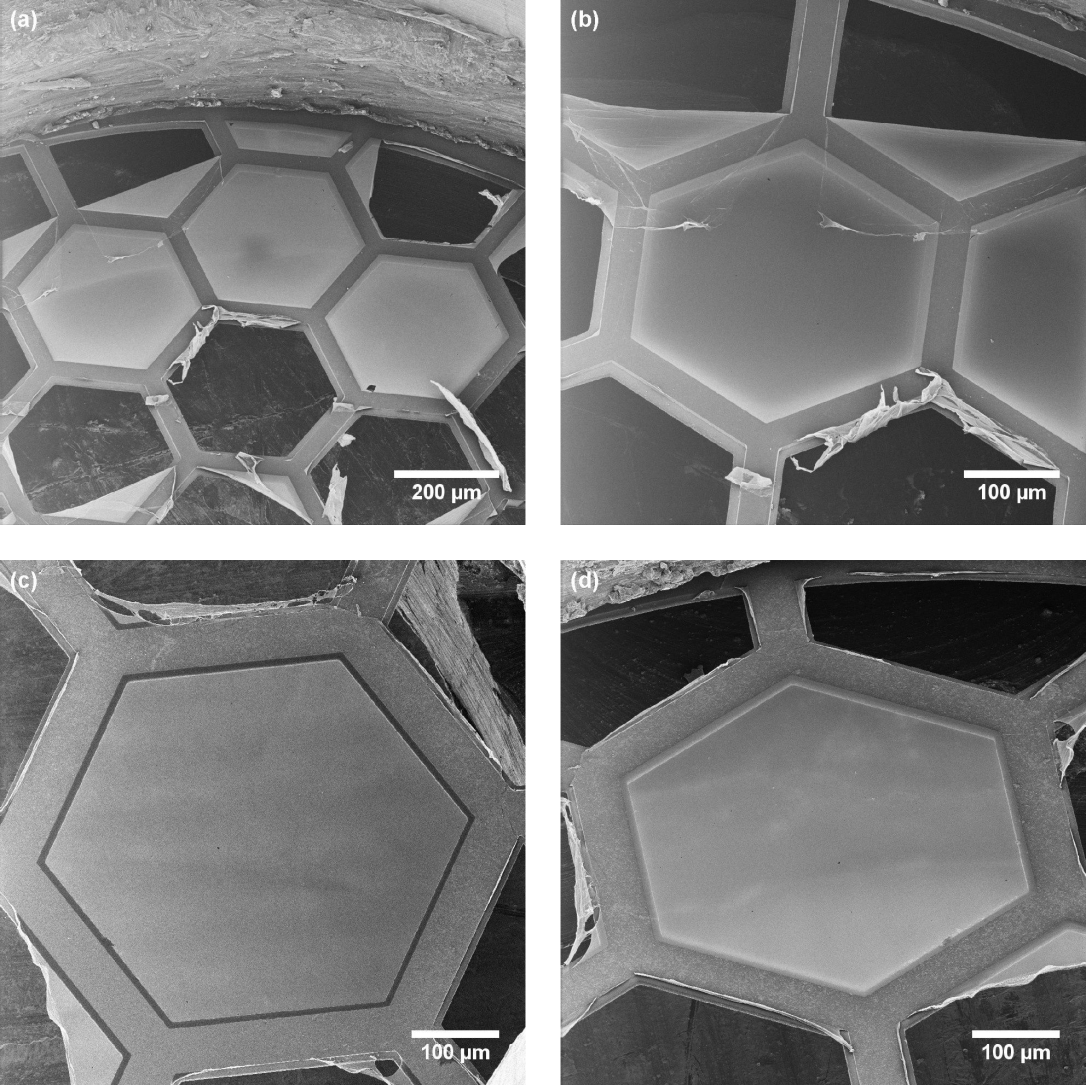
Examples of large freestanding CNMs. (a, b) Three intact CNMs are imaged at different magnifications. (c, d) Another intact CNM is imaged at different tilt angles. This comparison reveals that the intensity variation on the CNM surface originates mostly from secondary electrons emitted by the sample holder. Detailed information on all HIM images is given in Supporting Information File 1, Tables S1 and S2.

Examples of very large, freestanding CNMs are given in Figure 3. The ≈1 nm thin membranes are self-supporting over a distance of ≈0.5 mm, which are to date among the largest CNMs fabricated. The overview image in Figure 3a shows three intact freestanding CNMs, which are surrounded by ruptured membranes. In the upper part of this image, the sample holder is visible. The left membrane is shown with a higher magnification in Figure 3b. Apparently, the two ruptured membrane fragments in the upper part of the image are flipped over and cover part of the intact membrane. Another large CNM is presented in Figure 3c,d, which was imaged with different sample tilt angles. Note that the intensity variation on the CNM surface originates mostly from secondary electrons emitted by the sample holder as discussed earlier. This has been confirmed by varying the tilt angle of observation.

**Figure 4:**
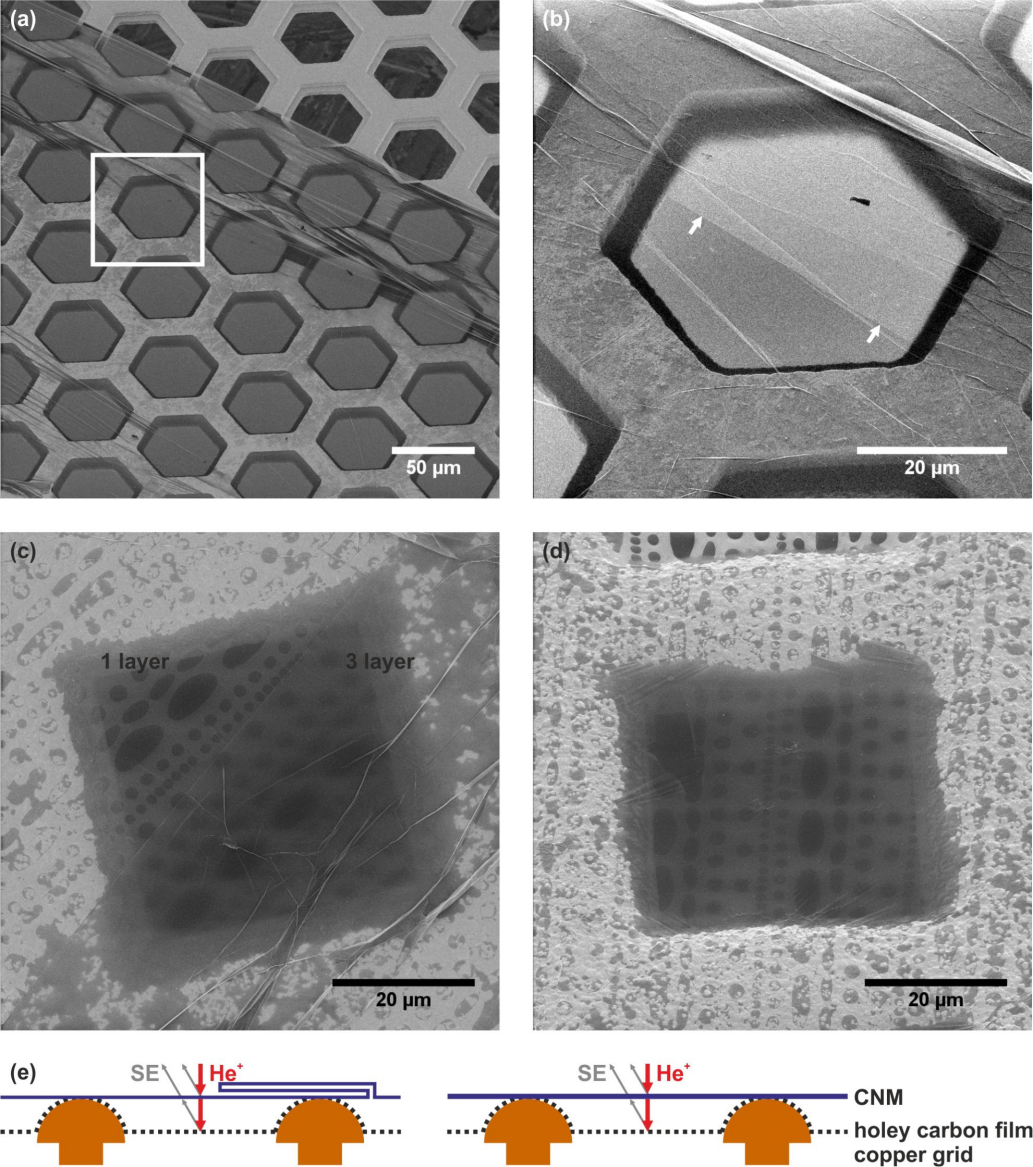
CNMs transferred on (a,b) bare copper TEM grids and (c,d) on grids with carbon films with regular openings (quantifoil multi-A). (b) A magnified image of the highlighted area in (a). (c,d) Increasing CNM thickness leads to more scattering of the He beam. The thickness of a single CNM layer is 1.1 nm in (c) and 1.7 nm in (d) [26]. (e) Schematic cross section of the samples in (c) and (d). The triple-layer region is folded according the scheme, which is consistent with the observed existence of a single-layer CNM on both sides of the fold. Detailed information on all HIM images is given in Supporting Information File 1, Tables S1 and S2.

CNMs with different thicknesses were also imaged in this study. An increased thickness provides more secondary electrons. This can be seen in Figure 4b, where part of a membrane was folded back onto itself, creating regions with double, triple and multilayers. In the overview image in Figure 4a, this double-layer region expands from the top left corner to the right middle of the image. The square marks the position of a magnified image (Figure 4b) where the start of the double layer region is marked with arrows. Note that the low contrast between single- and multi-layer CNMs in the overview image is related to charging. This was reduced in Figure 4b by employing the charge compensation system.

In Figure 4c,d the CNM is suspended on a copper grid with a holey carbon film some micrometres below it. A schematic cross section of both samples is depicted in Figure 4e. The CNM in Figure 4c is folded, so the lower right part is a CNM triple-layer with ≈3.3 nm thickness, where in the upper left part there is only one layer (≈1.1 nm). Figure 4d shows a CNM with 1.7 nm thickness. The structures of the holey carbon film become more blurred with increasing thickness due to an increase in scattering of the incident helium beam by the CNM before hitting the holey carbon film, as illustrated in Figure 4e.

**Figure 5:**
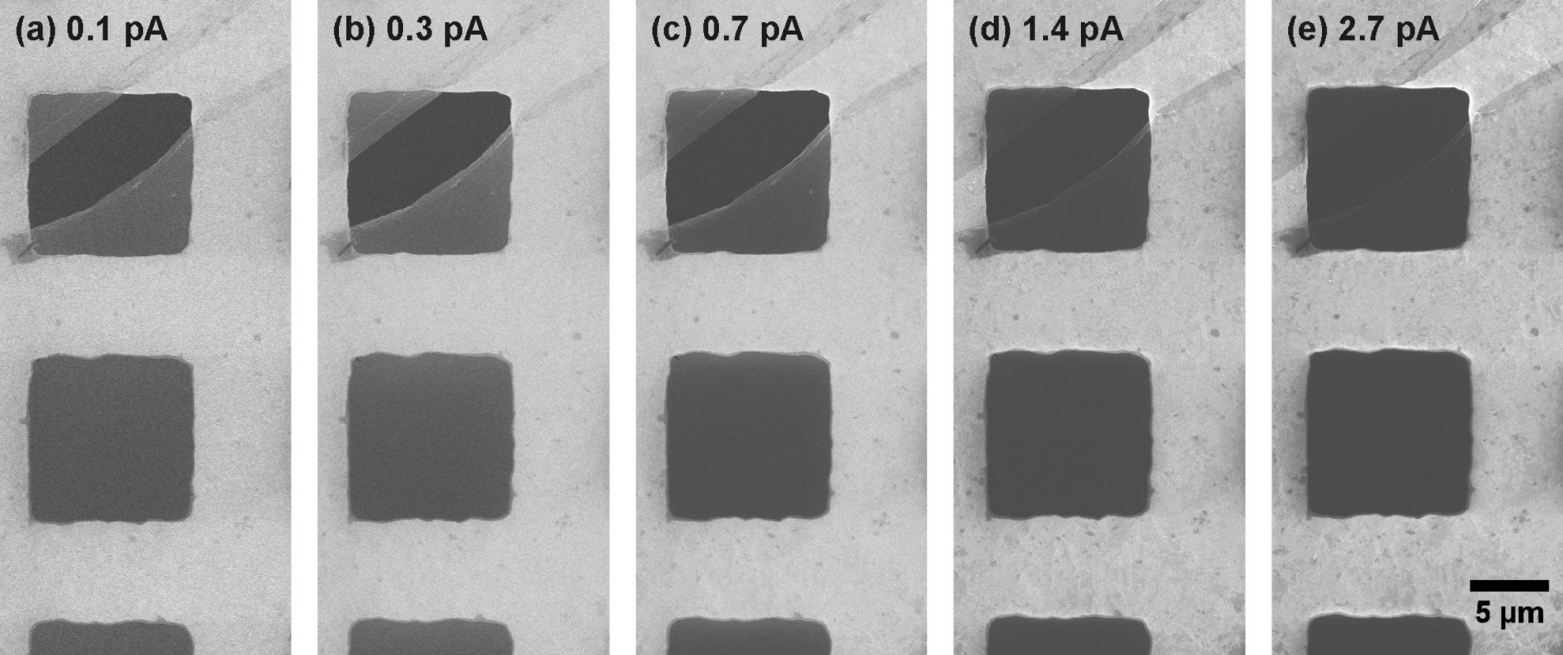
CNM on a gold grid. The same spot was imaged with different beam currents but under otherwise identical conditions. All images were taken without charge compensation. For a reasonable comparison of these images, the greyscale levels of the dark background of the uncovered openings as well as the surface of the bare gold areas were adjusted to be identical by means of changing the brightness and contrast of each whole image. Detailed information on all HIM images is given in Supporting Information File 1, Tables S1 and S2.

Figure 5 gives an image series that demonstrates the effect of charging. All images were recorded with a very low dwell time, maximum frame averaging, but without charge compensation and with different beam currents. The contrast and brightness settings were changed for each image in order to adjust the detector to a sensitive range. Each image in this series displays the identical sample position. The images show a gold TEM grid that is covered by a CNM. The upper, square opening exhibits a tear in the membrane from the upper right to the lower left corner. Each image shows 4 features of interest: intact, freestanding CNMs, CNM-covered gold surfaces, bare gold surfaces and the background, which is visible in the uncovered opening in the grid.

In Figure 5a–e an increase in the beam current is accompanied by an increase in the signal-to-noise ratio, which can be clearly seen on the bare gold surfaces. We also observed a darkening of the freestanding CNMs with increasing beam current due an increase in electrostatic charging. From this image series, we can determine the onset of charging for a 100 µm^2^ CNM under the aforementioned imaging conditions for beam currents of 0.3–0.7 pA. When imaged under the same conditions, membranes of the same size with a higher or lower conductivity should display an onset of charging at higher or lower beam currents, respectively. Thus, an imaging series such as that presented in Figure 5 might be able to give a comparative estimate about the conductivity differences between two membrane types. Note that differences in the secondary electron yield will also change the onset beam current for charging. Increasing the beam current not only leads to darker image areas for the freestanding membranes but also for CNMs on the gold support bars. Thereby, it increases the contrast between covered and bare gold surfaces.

**Figure 6:**
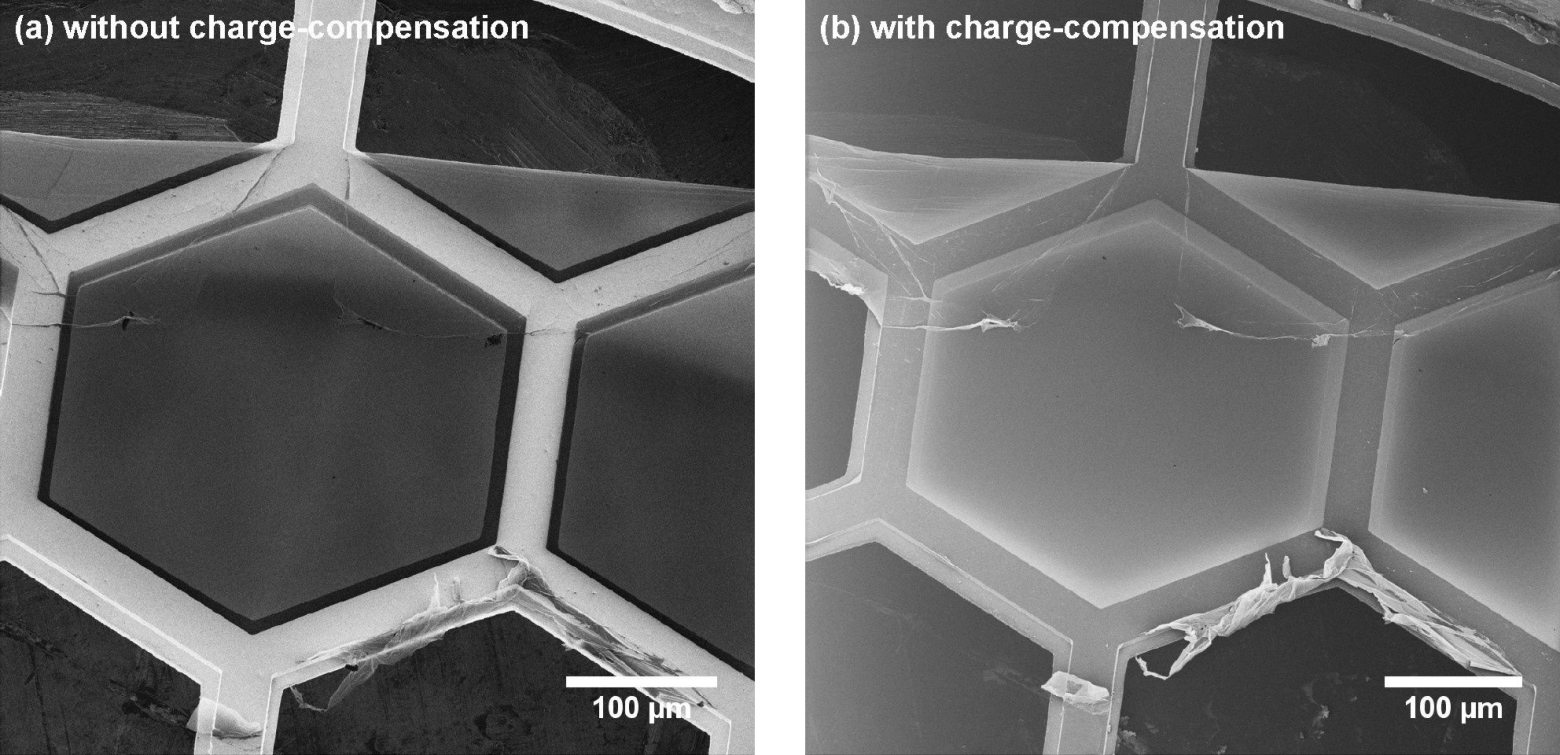
The same CNM is imaged (a) without and (b) with charge compensation. Detailed information on all HIM images is given in Supporting Information File 1, Tables S1 and S2.

The effectiveness of the electron flood gun for charge compensation in HIM is demonstrated by the images in Figure 6. A large area (i.e. ≈0.5 × 0.5 mm^2^), freestanding CNM is imaged without and with charge compensation in Figure 6a,b, respectively. As expected, the typical charged image features are removed when the compensation is employed. That is, the freestanding CNMs become brighter in comparison to the copper grid bars. A noteworthy observation in freestanding CNM regions close to the support structure can be made. Without charge compensation, these parts of the membrane appear darker than the central part of the CNM. This can be explained by the fact that near the support bar the CNM extents very close over the horizontal copper surface with a micron-sized gap. This is a result of the step-like shape of the supporting copper bars, which is illustrated in the grid cross section of Figure 1e. In the central part of the CNM, the He^+^ beam penetrates the membrane and impinges upon the sample holder underneath, which is far away and emits secondary electrons that reach the detector. Near the edges of the support, the He^+^ beam impinges on the step-like feature of the copper grid bar, which is very close to the CNM. Therefore, secondary electrons from this region are blocked by the CNM and do not reach the detector. Thus, in the central part of the CNM, a part of the detector signal originates from the sample holder, which is responsible for the observable inhomogeneous features in the CNM image. With charge compensation, as shown in Figure 6b, the membrane near the support structure becomes even brighter than the central part of the CNM. Again, the copper surface (some microns below the CNM) is responsible for this behaviour: Secondary electrons emitted from the copper reach the CNM and act as an additional charge compensation mechanism, leading to an increased signal at the detector. However, the charge compensation in the central part of the membrane is still sufficiently effective as the major signal originates from the CNM and not from the sample holder. This is quite obvious due to the increased image brightness as well as the more homogenous appearance of the central part of the CNM. Note that the described secondary-electron-induced partial charge compensation near the rim of CNMs also applies in Figure 6a, but in this case, the amount of secondary electrons emitted by all CNM areas is much lower than the contribution from the underlying sample holder.

HIM imaging of CNMs at higher resolution is possible in principle. However, homogenous CNMs do not possess any structures that can be imaged at the highest resolution of HIM. Such HIM images are featureless, showing only a constant grey value throughout the whole image (not shown here). This is different for CNMs with a structure imposed on the membrane. As an example, high resolution HIM images of CNMs are available where the membranes were exposed to highly charged ions [27]. This treatment induced nanopores in the size range of 10 nm, which were imaged by HIM with a reasonably high resolution [27]. Note that high resolution imaging of large freestanding CNMs requires the use of the electron flood gun for charge compensation as otherwise the membrane will easily rupture due to local charging.

## Conclusion

We have shown that helium ion microscopy is a very effective technique for characterizing CNMs. Additionally, CNMs have proven to be ideal test objects for evaluating the imaging characteristics of a HIM. The large range of magnification of a HIM allows for the visualization of TEM grids by recording a single HIM image. The effects of charging as well as background features were discussed. We demonstrated that the sample holder under the CNM surface can induce image artefacts, which are avoidable by mounting the grid on top of a deep cavity that acts like a Faraday cup. The presented systematic evaluation of membrane charging might enable the electrical conductivity of arbitrary 2D objects to be determined. The optimized HIM imaging of insulating membranes requires electron-flood-gun-based charge compensation, which was demonstrated with CNMs.

## Experimental

Helium ion microscopy (HIM) was performed with a Carl Zeiss Orion Plus^®^ microscope. The helium ion beam was operated at a current between 0.1–2.7 pA. The secondary electrons were collected by an Everhart–Thornley detector at 500 V grid voltage. For some images, the working distance was chosen to be as high as 37 mm, which allowed the acquisition of images with a very large field of view. The following imaging parameters were employed for optimized CNM imaging: a dwell time of 0.5 µs, up to 255 frame averages, and with the electron flood gun operated in line mode. The image acquisition was usually performed with fewer frame averages if the image noise level was observed to decrease to a negligible level.

The CNMs were prepared as described elsewhere [26] from the following molecules: (a) S-(pyren-1-ylmethyl) ethanethioate (MP1); (b) 1,1'-biphenyl-4-thiol (BPT); (c) S,S'-(3',4',5',6'-tetraphenyl-[1,1':2',1''-terphenyl]-4,4''-diyl) diethanethioate (HPB); (d) naphtalene-2-thiol (NPTH); (e) 2-bromo-11-(1’-[4’-(S-acetylthiomethyl)phenyl]acetyl)-5,8,14,17-tetra(3’,7’-dimethyloctyl)-hexa-*peri*-hexabenzocoronene (HBC-Br). The CNMs in the presented figures were produced from the following molecules: MP1 in Figure 1a,b; HPB in Figure 1d and Figure 2a,d; NPTH in Figure 2c and Figure 4a,b; HBC-Br in Figure 4c,d; and BPT in all other figures. As shown in Figure 4c,d, different SAM structures for HBC-Br molecules led to different CNM thicknesses [26]. In Figure 4c, one HBC-Br CNM layer is 1.1 nm thick and in Figure 4d, the thickness of the HBC-Br CNM is 1.7 nm.

## Supporting Information

File 1 Additional Experimental Information.
The supporting information provides details about the type of CNM and the employed HIM scan parameters for all presented images. Furthermore, exemplary SEM images of CNMs are shown.
