# Supplementary material for: Imaging of carbon nanomembranes with helium ion microscopy
Source: Beilstein J Nanotechnol. 2015 Aug 12;6:1712–20. doi: 10.3762/bjnano.6.175 (PMC4578422; doi:10.3762/bjnano.6.175)
Supplement: File 1 — Additional Experimental Information. The supporting information provides details about the type of CNM and the employed HIM scan parameters for all presented images. Furthermore, exemplary SEM images of CNMs are shown. [file Beilstein_J_Nanotechnol-06-1712-s001.pdf]

Supporting Information

for

Imaging of carbon nanomembranes with helium ion microscopy

André Beyer[1]*, Henning Vieker[2], Robin Klett[1], Hanno Meyer zu Theenhausen[1], Polina Angelova[2], and Armin Gölzhäuser[1]

Address: [1]Physics of Supramolecular Systems and Surfaces, Bielefeld University, 33615 Bielefeld, Germany and [2]CNM Technologies GmbH, 33609 Bielefeld, Germany

Email: André Beyer* - andre.beyer@uni-bielefeld.de

* Corresponding author

Additional Experimental Information

Table S1: Detailed information on all presented HIM images (Part 1 of 2).

Figure	CNM type[a]	CNM thickness[b] (nm)	Tilt angle (°)	Beam current (pA)	Dwell time[c] (µs)	Averaging mode	Number of averages[d]
1a	MP1	0.8	0	0.47	0.5	frame	≤255
1b	MP1	0.8	0	0.49	0.2	frame	≤255
1c	BPT	0.9	0	0.88	1.0	line	32
1d	HPB	0.8	35	0.28	0.5	frame	≤255
2a	HPB	0.8	35	0.18	1.0	frame	≤255
2b	BPT	0.9	45	0.3	0.5	frame	255
2c	NPTH	0.6	35	0.28	0.5	frame	≤255
2d	HPB	0.8	35	0.1	0.5	frame	≤255
3a	BPT	0.9	30	1.1	0.2	frame	≤255
3b	BPT	0.9	30	1.1	0.2	frame	≤255
3c	BPT	0.9	20	2.7	0.5	frame	≤255
3d	BPT	0.9	40	1.7	0.5	frame	≤255
4a	NPTH	0.6	35	0.28	0.5	frame	≤255
4b	NPTH	0.6	35	0.26	0.5	frame	≤255
4c	HBC-Br	1.1	0	0.37	0.5	frame	≤255
4d	HBC-Br	1.7	35	0.34	0.5	frame	≤255
5a	BPT	0.9	0	0.1	0.5	frame	255
5b	BPT	0.9	0	0.3	0.5	frame	255
5c	BPT	0.9	0	0.7	0.5	frame	255
5d	BPT	0.9	0	1.4	0.5	frame	255
5e	BPT	0.9	0	2.7	0.5	frame	255
6a	BPT	0.9	30	1.1	0.5	frame	≤255
6b	BPT	0.9	30	1.1	0.2	frame	≤255

[a]specifies the molecules used for the assembly of CNMs with the following names: (i) S-(pyren-1-ylmethyl) ethanethioate (MP1); (ii) 1,1'-biphenyl-4-thiol (BPT); (iii) S,S'-(3',4',5',6'-tetraphenyl-[1,1':2',1''-terphenyl]-4,4''-diyl) diethanethioate (HPB); (iv) Naphtalene-2-thiol (NPTH); (v) 2-Bromo-11-(1’-[4’-(S-Acetylthiomethyl)phenyl]acetyl)-5,8,14,17-tetra(3’,7’-dimethyloctyl)-hexa-peri-hexabenzocoronene (HBC-Br)

[b]values taken from [S1]

[c]dwell time corresponds to the time of uninterrupted recording of a single pixel which needs to be multiplied by the number of averages to yield the total exposure dose of each pixel

[d]the symbol “≤” indicates that in these cases the image acquisition was stopped at a lower number of averages as the image noise level had decreased to a negligible level

Table S2: Detailed information on all presented HIM images (Part 2 of 2).

Figure	Pixel size (nm)	Accelera-tion voltage (kV)	Aperture size (µm)	Spot control number[a]	Working distance (mm)	Flood gun mode[b]	Flood gun energy (eV)	Flood gun time (µs)
1a	1099	35.8	10	5	36.2	off	-	-
1b	1099	35.8	10	5	36.1	off	-	-
1c	1465	25.0	10	5	29.5	off	-	-
1d	317	35.8	10	5	21.2	frame	702	1000
2a	68	34.8	10	5	21.2	off	-	-
2b	98	15.0	20	6	26.8	off	-	-
2c	68	35.8	10	5	21.3	off	-	-
2d	73	34.8	10	5	21.4	off	-	-
3a	488	36.5	10	4	37.3	line	665	10
3b	269	36.5	10	4	37.3	line	665	10
3c	586	36.0	10	3.5	36.4	line	676	10
3d	586	36.0	10	4	37.5	line	676	10
4a	293	35.8	10	5	21.3	off	-	-
4b	68	35.8	10	5	21.3	frame	797	1000
4c	68	35.3	10	5	10.5	off	-	-
4d	68	35.1	10	5.4	21.1	off	-	-
5a	49	15	20	8	8.8	off	-	-
5b	49	15	20	7	8.8	off	-	-
5c	49	15	20	6	8.8	off	-	-
5d	49	15	20	5	8.7	off	-	-
5e	49	15	20	4	8.7	off	-	-
6a	244	36.5	10	4	37.3	off	-	-
6b	269	36.5	10	4	37.3	line	665	10

[a]this parameter adjusts the defocus of the He^+ beam at the beam limiting aperture

[b]line mode: charging is compensated between scans of individual lines; frame mode: charging is compensated between scans of individual frames

Imaging of CNMs with scanning electron microcopy

An example of scanning electron microscopy (SEM) images of CNMs is given in Figure S1. Both images show the same area on the same sample but with different contrast settings. A low acceleration voltage was chosen to improve the CNM contrast. However, in Figure S1a intact CNMs are nearly indistinguishable from ruptured membranes (e.g. the opening at the lower left image corner). Here the contrast setting allows imaging of the copper grid only. At higher contrast settings the detector is saturated by secondary electrons from the copper grid, i.e. these areas appear white in Figure S1b. On the other hand, intact CNMs are slightly brighter than ruptured CNMs, i.e. imaging of CNMs is possible by substantially increasing the contrast setting during SEM image acquisition. Thus, the low amount of secondary electrons emitted by CNMs makes it very challenging to characterize CNMs by SEM. Problems appear such as low signal-to-noise ratio, charging-induced rupturing at higher magnifications as well as the above described difficulty in setting the optimized contrast level. Note that this assessment is based on our experiences in imaging CNMs with a LEO 1530 field-emission SEM at 3 kV using the in-lens SE detector. Utilizing a suitable SEM with much lower acceleration voltages as well as a charge compensation system should allow CNM imaging with much higher quality.

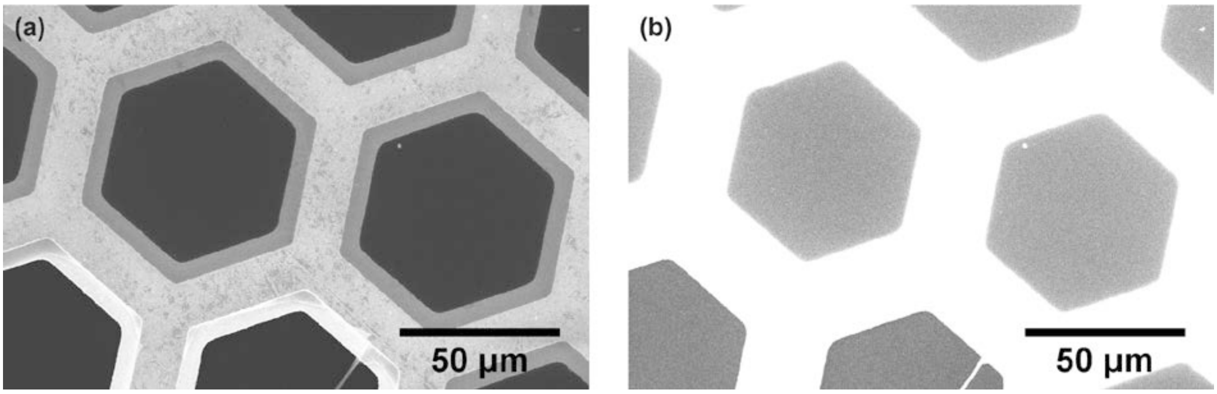

Figure S1: Scanning electron microscopy images of freestanding CNMs made from 1,1'-biphenyl-4-thiol (BPT) molecules. An acceleration voltage of 3 kV and a working distance of 8 mm was used. The sample was not tilted. Both images show the same area on the sample with (a) normal and (b) high contrast settings.

References

[S1] Angelova, P.; Vieker, H.; Weber, N. E.; Matei, D.; Reimer, O.; Meier, I.; Kurasch, S.; Biskupek, J.; Lorbach, D.; Wunderlich, K.; Chen, L.; Terfort, A.; Klapper, M.; Müllen, K.; Kaiser, U.; Gölzhäuser, A.; Turchanin, A. *ACS Nano* **2013,** *7* (8), 6489-6497.
